# Breaking Barriers Transforming Primary Care to Serve the Physical Health Needs of Individuals With SMI in the NHS

**DOI:** 10.1111/inm.13480

**Published:** 2025-01-23

**Authors:** Emma Sheridan, Oladayo Bifarin, Maria Caves, Karen Higginbotham, Jane Harris, Julie Pinder, Peter Brame

**Affiliations:** ^1^ Liverpool John Moores University Liverpool UK; ^2^ Edge Hill University Liverpool UK; ^3^ University of Bradford Bradford UK; ^4^ Merseycare NHS Foundation Trust Prescot UK; ^5^ British Society for Heart Failure London UK; ^6^ GM Stroke and Cardiac Network Liverpool Heart and Chest Hospital NHS Foundation Trust Liverpool UK; ^7^ University of Manchester Liverpool UK; ^8^ University of Salford Salford UK

**Keywords:** access, barriers, health inequalities, inequalities, physical health, severe mental illness

## Abstract

This critical review paper examines the health inequalities faced by individuals with Severe Mental Illness (SMI) in the United Kingdom; highlighting the disproportionate burden of physical health conditions such as respiratory disorders, cardiac illnesses, diabetes and stroke amongst this population. These conditions contribute to a significantly higher rate of premature mortality in individuals with SMI, with two‐thirds of these deaths deemed preventable. Despite the National Health Service (NHS) acknowledging the need to address these health inequalities, the mortality gap between those with and without SMI continues to widen. Additionally, there is limited engagement from service users in annual physical health checks, a concern that this paper addresses by identifying several barriers and providing recommendations to improve access and engagement in physical health checks. This review emphasises the focus on primary care systems as a critical point for addressing health disparities in individuals with SMI. Also, it highlights the need for primary care services to be more adaptive and integrated, playing a key role in managing the physical health of patients with SMI through regular health checks, flexible service delivery, and enhanced coordination with secondary care. Effectively supporting individuals with SMI requires tailored, integrated primary care interventions that address both psychological and physical health challenges, considering diverse demographic needs across the UK.

## 1 Aims

1. Identify existing barriers when attending or accessing support for physical health in primary care services;
2. Proffer recommendations to address these identified barriers; and
3. Articulate clear implications for clinical practice.

## 2 Background

The term SMI can include those diagnosed with bipolar disorder and schizophrenic disorders, among other chronic mental health disorders (Evans et al. 2016). In comparison to their counterparts, people living with SMI are disproportionally affected by chronic physical illnesses (Office for Health Improvement and Disparities 2023). It is common for those with SMI to live with co‐morbidities and multi‐morbidities which increases their physical health profiles (Murphy et al. 2018; NICE 2018). Consequently, individuals with SMI are likely to live 15–20 years less than the general population (Public Health England 2018; Walker, McGee, and Druss 2015).

The NHS is the publicly funded healthcare system in the UK, it provides comprehensive medical services to citizens for free. The NHS often produces its own guidance through governance papers and other guidelines. For instance, NHS England (2024) has recently published guidance on how to enhance the physical health care of those with SMI. They recommended 10 key actions which focus on; collaborative working with service users, multidisciplinary staff and carers, tackling health inequalities, personalised holistic care and addressing concerns of workforce and leadership (NHS 2024).

## 3 Design

This is a perspective paper that offers a critical review of factors perpetuating health inequalities of individuals with SMI with a specific focus on access to physical health care within primary care services.

## 4 Method

This paper utilised a non‐systematic review method, drawing from databases such as CINAHL, PubMed and grey literature, including NHS guidelines and health governance papers, to analyse barriers to accessing physical health care in primary care settings.

## 5 Physical Health of the SMI − Why Are They More at Risk?

Most antipsychotic and mood stabilising medications have long‐term effects on physical health (Correll et al. 2015; Murru et al. 2015). Crucially, some antipsychotics have been attributed to raising levels of prolactin, lipids, blood sugar, weight gain, and cardio‐toxic effects such as arrhythmias, myocarditis and cardiomyopathy (Li, Tang, and Li 2021; Annamalai 2017; Correll et al. 2015; Subramaniam et al. 2015; Scigliano and Gabriele 2013). Antipsychotics such as olanzapine, quetiapine, clozapine and risperidone and mood stabilisers such as lithium have also been associated with weight gain, chronic kidney disease and renal failure (Hedya, Avula, and Swoboda 2023; Dalal, Kar, and Agarwal 2022; Correll et al. 2015). Trying to meet the needs of service users living with SMI can be challenging and often requires the use of polypharmacy (Stassen et al. 2022). This creates risk of exposing service users to a greater amount, or exacerbating, side effects such as weight gain, cardiovascular issues, diabetes and parkinsonism (Annamalai 2017; Correll et al. 2015; Subramaniam et al. 2015; Scigliano and Gabriele 2013). In some instances, these side effects can be profoundly distressing and debilitating for their physical health (Stassen et al. 2022). This presents a challenging dilemma for prescribers and service users as the risk of developing such side effects and physical illnesses must be weighed against effective management of mental health symptoms (Pringsheim et al. 2017).

People living with SMI are statistically more likely to smoke, with suggestions that up to 50% of this population smoke in the UK (Gilbody et al. 2021). A multi‐national meta‐analysis study also highlighted that poor diet, poor sleeping patterns, limited exercise and drug and alcohol use are prevalent in this population (Vancampfort et al. 2019). Qualitative research within the UK specifically supports these claims (Carswell et al. 2022) and highlights that these could be attempts to cope with stressors. Wadsworth (2015) argues that while some coping strategies can be seen as maladaptive, it can also be understood as functional adaptations to adverse environments faced by individuals with SMI. Alternatively, research has suggested that some of these lifestyle factors are manifestations of SMI symptoms (Firth et al. 2020; Amodio et al. 2018). For instance, apathy and avolition can impact an individual's ability to partake in a healthy lifestyle (Amodio et al. 2018). Medication can also be a barrier, as many psychotropic medications harbour side effects such as cravings, weight gain and lethargy (British National Formulary, 2024) – meaning that it may be more difficult for this population to maintain a healthy lifestyle.

Socio‐economic factors such as low income, food insecurity and low levels of health literacy are also closely associated with unhealthy lifestyle choices (Pechey and Monsivais 2016; Raphael 2016). A multi‐cohort study of Finnish and UK data found that low socioeconomic status heightened the risk of 18 out of 56 health conditions leading to physical illnesses like liver disease, cardiovascular disease, and dementia (Kivimäki et al. 2020). Similarly, a cohort study in Northern Ireland also found higher associations between SMI and social deprivation (McCarter et al. 2023). These findings emphasise addressing mental health in a social context to reduce health inequalities.

This research suggests that the poorer physical health of individuals with SMI is due to multiple factors. Nonetheless, it is crucial to support them in understanding their risk of physical health co‐morbidities, particularly since many have low health literacy (Raphael 2016; Degan et al. 2021). Healthcare providers should therefore consider this and implement targeted health literacy interventions.

Health guidelines in the UK endorse a number of lifestyle change interventions such as the use of brief verbal interventions, goal‐setting and motivational interviewing (NHS 2019a, 2019b; NICE 2014). However results from a mixed‐methods pilot study questions the effectiveness of these interventions − specifically motivational interviewing was found to have limited effect on SMI (Kirschner et al. 2022). Degan et al. (2021) argues that there are limited studies on engaging SMI in their physical health and more research is needed to develop successful health change interventions for SMI specifically (Degan et al. 2021).

Regardless of its causes, SMI premature mortality remains a major health inequality in modern society. Hence, this continues to be frequent on UK healthcare agenda (NHS England 2024; O'Neil, Heenan, and Betts 2019; Public Health England 2018; NHS England 2018; Scottish Government 2017; Mental Health Taskforce 2016). Despite this awareness, the mortality gap between people with SMI and people without SMI continues to widen (Byrne 2023). Alarmingly, most of the co‐morbidities and premature mortalities in SMI populations are considered preventable (McCarter et al. 2023; Janssen et al. 2015), and could have been addressed by more rigorous physical health assessment and intervention from HCPs (McCarter et al. 2023).

## 6 Double Jeopardy − Health Inequalities in Minoritised Ethnic Groups With SMI

The idea of “Double Jeopardy” describes the compounded health risks for individuals with SMI from minoritised ethnic groups, who face both SMI‐related health disparities and additional barriers due to systemic inequalities and cultural factors, leading to worse health outcomes and higher rates of chronic conditions. Ethnicity is a contributing factor to the development of adverse health outcomes. For example, a non‐systematised review found that non‐white individuals are 3–5 times more vulnerable to acquiring preventable medical illnesses such as diabetes in England and Wales (Goff 2019). Similarly, a cohort study in England found that Black women with SMI were almost twice as likely to experience multi‐morbidities compared to their white counterparts (Catalao, Dorrington, and Pritchard 2022).

Minoritised ethnic groups also have poor rates of engagement with health services often only presenting when their illness has reached a critical stage and therefore may no longer be preventable (Bansal et al. 2022). Lack of engagement is particularly prevalent in Black males (Memon et al. 2016). Qualitative research has found that a lack of trust in healthcare services is also disproportionately low in minoritised ethnic groups and likely a key contributor to the lower levels of health‐seeking behaviour (Phillips 2022). Consequently, minoritised ethnic individuals with SMI are subjected to a double jeopardy of health inequalities.

It is also essential to consider that minoritised ethnic groups encompass different cultures and ethnic backgrounds. Amongst research in the UK, there is a tendency to cluster all non‐white individuals into one homogenous group (Ojo‐Aromokudu et al. 2023). This generalises behaviours and disease prevalence between each of these ethnicities (Ojo‐Aromokudu et al. 2023). Future research on SMI therefore would benefit to acknowledge the individual differences within minoritised ethnic groups rather than generalising them collectively (Ojo‐Aromokudu et al. 2023).

## 7 The Critical Role of Primary Care in Integrating Physical and Mental Health Services for SMI

Within the NHS, care of those with mental health conditions is managed at two levels through either primary or secondary care. Primary services consist of services such as General Practitioners (GP) and pharmacies. Secondary care services often require referrals and consist of services such as community mental health teams, inpatient units and specialist physical health services. Traditionally, primary and secondary services have been distinctly separate and operated in vastly different ways (Rodgers et al. 2018). Collaboration between services was often lacking and each service operated within its own specialty (Rodgers et al. 2018).

The Care Programme Approach (CPA) was introduced in 1991 with the aim to ensure that individuals with complex SMI presentations were provided with a safety net of care (Kingdon 2019). This entailed regular assessments with their consultant psychiatrist, having an individualised care plan and ongoing support from a Community Psychiatric Nurse (CPN) (Kingdon 2019). While the effectiveness of CPA has been debated, it provided benefits such as regular evaluation of psychotropic medications, protecting vulnerable service users from slipping through the net, and providing CPN assistance with navigating healthcare systems and networks (Simpson, Miller, and Bowers 2003; Bartels et al. 2013; van Hasselt et al. 2013).

The CPA framework was subsequently replaced by the Community Mental Health Framework (NHS 2019a, 2019b), which promotes the integration of care between primary and secondary services, and emphasises a shared responsibility for managing the physical health of individuals with SMI. Despite this shift, it is evident that General Practitioners (GPs) continue to act as the primary gatekeepers to secondary physical health services (Sripa et al. 2019). This poses a challenge, as people with SMI can often have poor engagement with primary care services (Parker et al. 2023).

Across the UK, beyond ad‐hoc appointments, physical health monitoring for individuals with SMI is mandated through annual reviews (NHS 2019a, 2019b). Current guidelines state that it is primary care's responsibility to undertake physical health checks for service users with SMI who are not in mental health crisis and not open to or new (less than a year) to secondary mental health services (NHS 2019a, 2019b). Additionally, GPs receive financial incentives from Integrated Care Boards (ICBs) upon completion of these checks (NHS 2019a, 2019b). However, for service users open to both primary and secondary care, data sharing on physical health checks have been reported to be poor, resulting to duplicate or incomplete physical health assessments (McGuinness and Follan 2016).

A five‐year cross‐sectional study, covering 90% of GP services in England, found that the average uptake of physical health checks in the general population was 52.6%, (Patel et al. 2020). Whereas, the uptake for individuals with SMI in primary care during the third quarter of 2021/2022 was just 34.9% (NHS 2022). This significant disparity highlights the urgent need to identify and address barriers to care access and to implement targeted interventions for individuals with SMI. Similarly, uptake is low across Northern Ireland and Scotland (McCarter et al. 2023; Scottish Government 2017). This research demonstrates that critical action is needed from primary care to enhance the re‐uptake of physical health checks.

## 8 Barriers to Health Equity for Severe Mental Illness in Primary Care

The symptomology of SMI is a challenge when considering access to services. Symptoms such as disorganised thinking, poor planning and avolition can contribute to appointment non‐adherence (Melamed et al. 2019). A qualitative study in Belgium found some service users experience difficulties in arranging and remembering appointment dates as well as planning or financing their travel route to GP surgeries (Kohn et al. 2022). Individuals with SMI are also more at risk of fatigue and psychological burnout resulting from their illnesses and as a side‐effect of medication (Ince, Partlak, and Serçe 2019). These symptoms can often contribute to appointment cancellations or non‐attendance (Gedik, Partlak Günüşen, and Çelik Ince 2020).

Side effects of psychotropic medications are further associated with difficulties in implementing health promotion advice (Ho et al. 2022). Service users report great difficulty in trying to maintain a healthy lifestyle due to increased appetite and weight from medications (Ince, Partlak, and Serçe 2019). This is so pronounced that some service users consider attending appointments for the management of physical health as futile due to HCP's unrealistic expectations, lack of understanding around difficulties associated with symptomology (such as lethargy and apathy) and limited knowledge of how side effects of medications can affect their physical health (Ho et al. 2022; Björk Brämberg et al. 2018; Lamontagne‐Godwin et al. 2018).

Stigma is a significant barrier to accessing and engaging with physical health checks and treatment. A systematic review of 24 multi‐national studies by Hallyburton and Allison‐Jones (2023) revealed that 5 studies identified that perceived stigma from primary care HCPs deters service users from accessing their GP. Qualitative research in the UK and beyond showed that service users attribute feeling disempowered when making choices about their own health because of this stigma and the perceived hierarchy between GPs and themselves (Thomas et al. 2023; Ho et al. 2022; Gedik, Partlak Günüşen, and Çelik Ince 2020; Lavie‐Ajayi et al. 2018; Ehrlich and Dannapfel 2017).

There are also cases of diagnostic overshadowing, where HCPs attribute service users' descriptions of physical health ailments to be symptoms of their mental health diagnosis (Hallyburton and Allison‐Jones 2023). Diagnostic overshadowing is considered both as having an impact on the quality of physical health assessments and as a barrier for service user engagement when they were aware of this occurring (Hallyburton and Allison‐Jones 2023; Kohn et al. 2022; Björk Brämberg et al. 2018).

Qualitative research in the UK has further identified that individuals with an SMI are perceived by some HCPs as non‐adherent to care plans and unwilling to engage with primary care (Carswell et al. 2022). Such preconceptions have perpetuated feelings of de‐motivation and diminished confidence when working with these service users, this is particularly exacerbated for HCPs with less experience of working with SMI (Ho et al. 2022). Qualitative research has identified that several HCPs also report fear of being transparent with service users concerning the associated risks with medications, as they anticipated higher levels of non‐concordance (Bressington et al. 2018). A recent integrative review identified three studies whereby carers of SMI felt that HCPs sometimes did not address side effects and co‐morbidities out of fear of mental health deterioration if they changed the treatment plan (Ho et al. 2022). Similarly, the qualitative study by Carswell et al. (2022) found that healthcare professionals and carers in the UK are acutely aware that the management of mental health is prioritised over addressing physical health decline. This treads an ethical dilemma whereby HCPs may believe that they are safeguarding the service user by preventing relapse, but they are failing to consider the rights and freedoms of choice of the Human Rights Act (1998) as well as diminishing service users' quality of life.

## 9 Bureaucracy at Play − Primary Care Services

Debate is ongoing regarding whether HCPs with mental or physical health backgrounds are better equipped to perform the annual physical health checks for SMI. Literature suggests that RMNs (Registered Mental Health Nurses) lacked robust physical health training and were not adept to effectively manage or interpret physical health data (Lamontagne‐Godwin et al. 2018; McGuinness and Follan 2016; Robson et al. 2013). Likewise, RGNs (Registered General Nurses) and other primary care practitioners were associated with poorer knowledge of SMI and limited understanding of how to support or motivate these service users (Ho et al. 2022). This indicates how further training for all HCPs is needed regardless of discipline.

Interestingly, a quasi‐experimental inpatient study in England employed the use of enhanced staff training, patient education and a patient‐held physical health record. The uptake of physical health assessments increased by 15.6% (Green et al. 2018). This suggests that staff training and collaboration with the patient can achieve positive results. It would be interesting if future research could test the reliability of these findings in primary care settings.

High staff turnover of staff also results in inconsistent service provision, which impedes continuity of care in terms of having familiar HCPs who know the service user well (Melamed et al. 2019). For service users with SMI, the importance of this cannot be overstated, as many of them report frustrations about having to re‐tell their experiences to several health professionals (Biringer et al. 2017), which can be difficult for individuals with an SMI and further compound issues relating to sustaining trusting and therapeutic relationships (Biringer et al. 2017). In essence, when service users are aware that their annual physical health check is with an unfamiliar HCP, they may disengage (Small et al. 2017).

Limited flexibility within GP processes continues to hinder engagement, with research showing that many individuals with SMI are more likely to attend later appointments (Melamed et al. 2019; Gronholm et al. 2017; Nankivell et al. 2013; van Hasselt, Oud, and Loonen 2013). However, these are not always available due to the current structure of GP services. Existing literature argues for more flexible approaches (Musiat and Tarrier 2014; Voort et al. 2022; Hallyburton and Allison‐Jones 2023); by offering varied appointment times, considering the use of mobile sites such as those used in breast screening services and offering home visits (Musiat and Tarrier 2014; Gronholm, Onagbesan, and Gardner‐Sood 2017; Voort et al. 2022; Hallyburton and Allison‐Jones 2023).

## 10 Effective Integrated Care − A Need to Respond and Coordinate Tailored Care

The collaboration and integration of primary and secondary mental health services is a critical component in the future of healthcare delivery (NHS 2019a, 2019b). Organisations working closer to offer continuity of care have better health outcomes, particularly for individuals with mental health diagnoses (Jeffers and Baker 2016). Despite advancement in integrating existing services, it remains a major challenge in healthcare. For instance, data sharing is still poor across services (Kohn et al. 2022; Lerbaek et al. 2021; Ho et al. 2022; Melamed et al. 2019; Gronholm, Onagbesan, and Gardner‐Sood 2017; McGuinness and Follan 2016; Ehrlich et al. 2014). This increases frustration with service users as this results in needless duplication of appointments, patients having to recount their history and repeated testing, which wastes resources and time (van Hasselt, Oud, and Loonen 2013).

There is a need for services to be more assertive to seek opportunities for better integration and collaboration in practice (Lerbaek et al. 2021). This may however be challenging when considering that there still remains limited understanding as to how each service operates in the eyes of the other (Bressington et al. 2018; Lamontagne‐Godwin et al. 2018). Whilst training may provide some benefit, it is clear that services differ significantly in their bureaucratic processes and priorities that they continue to operate, for the most part, distinctly and are not financially incentivised to work together.

Individuals with SMI require a support network to help them attend appointments (Melamed et al. 2019). Family, care coordinators, and carers are often relied upon to help remember appointments, encourage attendance, arrange transport and attend these appointments with them (Ho et al. 2022; Melamed et al. 2019). This is not always feasible as a significant proportion of the SMI population has a limited support network (Gedik, Partlak Günüşen, and Çelik Ince 2020). Similarly, due to the strain on staff in the social and health sector, staff also are not always available to attend to the service user (van Hasselt, Oud, and Loonen 2013; Nankivell et al. 2013). The crowded and busy nature of GP surgeries can be intimidating for those with SMI (van Hasselt, Oud, and Loonen 2013, 2015; Melamed et al. 2019), therefore having a known person to provide emotional supporting during this may be beneficial. For those who experience high levels of social anxiety it may also be challenging for them to have to travel on public transport alone to attend surgery (Conceição et al. 2023). Therefore, if there are no other means of transport accessible to them, they may not attend. Consideration also needs to be given to avoid the burnout of carers (NHS 2024). Hence, health systems need to be more accommodating for these service users (Gedik, Partlak Günüşen, and Çelik Ince 2020). Alternatively, hope could be found in the introduction of outreach teams which some localities already are incorporating across the UK (NHS England 2023). Whilst there is limited evidence of the efficacy of outreach teams specifically for SMI in primary care, studies have shown that outreach teams can enhance engagement by up to 87% (Carpenter, Luce, and Wooff 2011).

The international council of nurses proposes the use of care navigators to help alleviate these issues (ICN 2024). A scoping review of the use of care navigators in the mental health systems in Canada yielded positive results (Mullen, Levitt, and Markoulakis 2022). However, more is needed to assess its efficacy for assisting the SMI navigating physical health services in the UK.

## 11 Discussion

This paper has examined the physical health care received by people living with SMI in the UK, the role of primary and secondary care HCPs within this and interrogate the issues relating to existing systems and processes. Findings from this paper reiterates that individuals living with SMI continue to experience health inequalities when trying to access support for their physical health. Perceived stigma from HCPs, symptomology, side effects of medication, poor rapport with staff, diagnostic overshadowing, inflexible timings of appointments and limited support to accessing these appointments were associated with non‐attendance and poor engagement at annual physical health appointments (Melamed et al. 2019; Ho et al. 2022; Ehrlich and Dannapfel 2017).

This paper has highlighted the need for specific interventions and minimised stigma when treating the SMI. There is a need to be more transparent about the potential risks psychotropic medication and lifestyle poses on mental health (Bressington et al. 2018). There is also a need for greater flexibility with the timings and locations of appointments (Voort et al. 2022), and consideration into employing staff specific to supporting coordination and subsequent actions of physical health checks (ICN 2024; Björk Brämberg et al. 2018; Mental Health Foundation 2013).

There remains disputes as to whether RGNs or RMNs are better suited to complete physical health checks for the SMI (Ho et al. 2022; Lamontagne‐Godwin et al. 2018; McGuinness and Follan 2016; Robson et al. 2013). Regardless of discipline, the literature is unified in its recommendations for better training opportunities for each of these HCPs to address these knowledge deficits, minimise stigma, diagnostic overshadowing and share strategies to help better engage service users with their physical health (Hallyburton and Allison‐Jones 2023; Kohn et al. 2022; Lamontagne‐Godwin et al. 2018; Björk Brämberg et al. 2018).

We argue that existing funding mechanisms in the UK's healthcare system create financial disincentives for adopting innovative practices across all levels of care, thereby maintaining the status quo and limiting progress in improving health outcomes. As such, NHS Confederation asserted that, financial flows within the NHS are fragmented and hinder integration. The various components of the NHS − primary care, community care, and hospital care − lack financial incentives to collaborate more effectively (Jones, Williamson, and Barron 2024).

The literature is unified in suggesting that more research needs to be done to better understand and address barriers and health inequalities experienced by those with SMI (Rodgers et al. 2018; Public Health England 2018; Mental Health Foundation 2013). However, despite aforementioned guidance, concerns remain that nurses, who are at the heart and forefront of the NHS, are no longer actively nurtured to conduct research (McKenna and Thompson 2024). Arguably, increased pressure on the NHS workforce is stifling opportunities for research (Toh and Haynes 2022), and the shift in university paradigms to favour teaching rather than research further harbours the progression of research (McKenna and Thompson 2024). This is further compounded by a binary approach limits the potential of educational frameworks by adhering too rigidly to either maintaining the status quo or promoting radical change, without adequately addressing the nuances and complexities of nurse education (Collier‐Sewell and Monteux 2024). All of these have detrimental impacts on the existing health system, as it risks ignoring opportunities for innovation and becoming outdated in its practice. NHS (2024) guidelines have made it a priority for research opportunities to be available for HCPs and for better collaboration with established researchers and Higher Education Institutions (HEIs). HCPs especially mental health nurses who are better positioned to care for people living with SMIs must make conscious effort engage in research activities to take control over their professional identity and address the historical shortfall of mental health research (NHS 2024; Meehan and Robertson 2012).

It is also important to consider that, despite movement towards integration, the culture of physical and mental health being distinct disciplines remains (Trenoworth 2022). Whilst it is essential to acknowledge the specialised knowledge and skills each discipline provides and avoid the dilution of these roles (Bifarin et al. 2024), it remains a paradox that whilst this culture remains it may be challenging to persuade staff to value the importance of roles which incorporates skills from both disciplines. However, as integration of services is still in an early phase the future may allow opportunities for more roles which incorporate enhanced knowledge in both physical and mental health, care navigation and better communication between primary and secondary services. It is reassuring to see some movement towards integrated working; by some PCN (Primary Care Networks) employing mental health nurses and likewise some mental health teams employing physical health nurses (NHS England 2016).

## 12 Recommendations for Clinical Practice

Based upon the findings, this paper proposes the following recommendations:

1. There is an urgent need to have a more integrated service between primary care and mental health services to avoid duplication of work and promote better understanding of the physical health of people with SMI and help them navigate the health system. The greatest challenge is addressing inequalities at local level. This is due to each region of the UK being comprised of different ethnic‐socio‐economic populations which presents diverse pressures, complexities, and demand for each individual trust and primary care networks. Therefore, work on integration should be considered within the context of locality with support from local authority.
2. A need for educational change; to upskill primary and secondary care staff with role‐specific training. In this, it is also important to consider integrating the physical health of SMI more into teaching of pre‐registration nursing. It is essential that the future nursing workforce is prepared adequately for integrated working. Whilst it is difficult to prepare for the uncertainties in the future of the NHS, student nurses need to be prepared to thrive in uncertain and complex environments. This involves encouraging critical thinking, practical reasoning, and the ability to deal with incomplete and evolving knowledge.
3. There is an urgent need to shift organisational cultures within primary and mental health services which often overemphasise metrics and administrative tasks, underpinned by various bureaucratic processes, to recovery‐oriented care provision. This will be of great relevance to stress alleviation for staff, in regard to administrative tasks and role confusion among nurses. This should assist in retention and increase capacity to provide more effective and tailored care.

## 13 Conclusion

Individuals with SMI continue to face significant disparities in their physical health care. This paper has identified complex factors that influence service users' engagement with primary care. Key issues include fragmented organisational and financial structures within the NHS that discourage integrated care, the lack of support in navigating the health system and the pervasive stigma associated with SMI that hinders effective care delivery.

The literature consistently recommends enhancing training for HCPs to address knowledge deficits, reduce stigma and diagnostic overshadowing, and to adopt strategies that improve engagement with service users.

Moreover, the current distinction between physical and mental health disciplines within the NHS remains a significant barrier to holistic care. Moving forward, the integration of services, supported by stronger collaboration between academic institutions and healthcare trusts, is essential. Such integration could foster more roles that encompass both physical and mental health care skills, promoting better communication and coordination between primary and secondary care services. Ultimately addressing these health inequalities requires systemic changes in policy, practice, and education. By implementing these changes, the NHS can enhance patient care, reduce premature mortality rates, and ensure that individuals with SMI receive the comprehensive care they deserve.

## 14 Relevance for Clinical Practice

This paper provides a contemporary examination of the health inequalities that those with SMI experience when accessing support for their physical health. It highlights the practical and bureaucratic challenges that these service users experience in the context of the NHS in the United Kingdom. It provides recommendations for NHS services as well as HEIs to tackle these health inequalities, with emphasis on enhancing collaborative practice to improve care.

## Author Contributions

Each of the authors listed meet the criteria for authorship in accordance with International Committee of Medical Journal Editors by each being involved in the conceptualisation, drafting stages and final approval of the work.

## Conflicts of Interest

The authors declare no conflicts of interest.

## Data Availability

Data sharing not applicable to this article as no datasets were generated or analysed during the current study.
